# Infliximab Dose Reduction Sustains the Clinical Treatment Effect in Active HLAB27 Positive Ankylosing Spondylitis: A Two-Year Pilot Study

**DOI:** 10.1155/2013/289845

**Published:** 2013-09-05

**Authors:** Boel Mörck, Rille Pullerits, Mats Geijer, Tomas Bremell, Helena Forsblad-d'Elia

**Affiliations:** ^1^Department of Rheumatology and Inflammation Research, The Sahlgrenska Academy at Gothenburg University, Guldhedsgatan 10A, 41346 Gothenburg, Sweden; ^2^Department of Radiology, Sahlgrenska University Hospital, Bruna stråket 11B, 41345 Gothenburg, Sweden; ^3^Center for Medical Imaging and Physiology, Skåne University Hospital, Lund University, 22185 Lund, Sweden

## Abstract

The rationale of the study was to evaluate the efficacy of infliximab (IFX) treatment in patients with ankylosing spondylitis (AS) and to determine whether IFX dose reduction and interval extension sustains the treatment effect. Nineteen patients were included and treated with IFX 5 mg/kg every 6 weeks for 56 weeks. All patients concomitantly received MTX with median dose 7.5 mg/weekly. During the second year, the IFX dose was reduced to 3 mg/kg every 8 weeks. Eighteen patients completed the 1-year and 15 patients the 2-year trial. The ≥50% improvement at week 16 from baseline of BASDAI was achieved in 16/19 (84%) patients. Significant reductions in BASDAI, BASFI, and BASMI scores, decrease in ESR and CRP, and improvement in SF-36 were observed at weeks 16 and 56. The MRI-defined inflammatory changes in the sacroiliac joints disappeared in 10/15 patients (67%) already at 16 weeks. IFX treatment effect was sustained throughout the second year after IFX dose reduction and interval extension. We conclude that IFX treatment is effective in well-established active AS and a dose reduction sustains the treatment effect. These observations are of clinical importance and open the opportunity to reduce the drug costs. This trial is registered with ClinicalTrials.gov NCT01850121.

## 1. Introduction

Ankylosing spondylitis (AS) is a chronic, progressive inflammatory disease that primarily affects the spine and sacroiliac joints. The disease has a prevalence of about 0.55% of the European population [1, 2] and is closely associated with HLA-B27 positivity. The disease affects mostly young individuals in the third and fourth decade of their life and may therefore have a major impact on their work ability, which is associated with increased costs to the patient and the healthcare system [3].

Disease modifying antirheumatic drugs (DMARDs), including methotrexate and sulfasalazine, have not shown efficacy in treating the axial manifestations of AS but may be beneficial in treating peripheral joint disease [4]. NSAIDs along with patient educational programs, regular physiotherapy, and exercises have been recommended as the standard therapy for axial AS. TNF-alpha antagonists have made it possible to notably improve the health status in AS patients. The efficacy of TNF antagonists has been demonstrated in several short-term clinical studies [5] as well as in long-term studies [6–8]. Although compelling data is increasing indicating that infliximab is effective for treatment of AS, most randomized, placebo-controlled studies have evaluated a treatment dose of 5 mg/kg every 6 weeks. A few reports have been published showing that infliximab in a low-dose regimen (3 mg/kg) is also effective in suppressing signs and symptoms of active AS [9–15]. However, the need for dose escalation up to 5 mg/kg due to partial treatment effect has been reported highly varying in different study cohorts [13–15]. Dose escalation was necessary in 15% and 18% of patients as reported by Maksymowych et al. [10] and Jois et al. [14], respectively. In contrast, in two other studies it was found that 61%–63% of patients required dose escalation [12, 15]. However, it is currently unknown whether the treatment effect achieved with a dose of 5 mg/kg of infliximab every 6 weeks is maintained after dose reduction to 3 mg/kg every 8 weeks. 

The initial objectives of the current study were to evaluate the efficacy of infliximab (5 mg/kg) treatment on the clinical disease activity, MRI assessed inflammatory changes in the sacroiliac joints and quality of life in patients with HLA-B27 positive active AS at 16 and at 56 weeks. Additional objective of importance was to determine whether infliximab dose reduction to 3 mg/kg every 8 weeks during second year would retain the treatment effect.

## 2. Materials and Methods 

### 2.1. Patients and Study Protocol

Twenty-three consecutive patients with active AS identified at the Department of Rheumatology Outpatient Clinic, Sahlgrenska University Hospital, Gothenburg, during the period of June 2003 to November 2006, were invited to participate in the study. The diagnosis in each patient had been made prior to the study by the treating rheumatologist (Boel Mörck). None of the patients had received previous treatment with biological agents. The patients had to fulfill the following four inclusion criteria: (I) age between 18 and 60 years, (II) proven diagnosis according to the modified New York criteria [16] for definitive AS, (III) active disease with Bath AS Disease Activity Index (BASDAI) score ≥4, and (IV) current or previous treatment with conventional nonsteroidal anti-inflammatory drugs (NSAID) in adequate doses without sufficient effect. 

Exclusion criteria were as follows: current signs or symptoms of severe, progressive, or uncontrolled hepatic, hematological, pulmonary, cardiac, neurological, or cerebral disease; ongoing or past serious infection (including HIV and past or current tuberculosis); pregnancy or breast feeding; current malignancy or history of malignancy within the past five years; congestive heart failure; any contraindication to MRI. 

This study was approved by the Regional Ethics Committee in Gothenburg and an acceptance was obtained from the Medical Product Agency since infliximab was not approved for treatment of AS at the commencement of the study. The study was performed in accordance with the Declaration of Helsinki and informed consent was obtained from all patients.

### 2.2. Treatment Protocol

The patients fulfilling the inclusion criteria were treated with intravenous infusion of infliximab (5 mg/kg) at week 0, week 2, and week 6 of the study and thereafter every 6 weeks for a total of 56 weeks. All patients were concomitantly treated with methotrexate. DMARDs other than methotrexate were not allowed and were discontinued at least 4 weeks prior to inclusion. Treatment with NSAIDs and methotrexate (median dose 7.5 mg/weekly) remained unchanged or a dose reduction was allowed during the study period. Initiation of oral corticosteroids was not permitted during the study. After having completed the first year, the dosage of infliximab was reduced to 3 mg/kg and median infusion interval extended to every 8 weeks during the second year. Patients were thereafter followed up on a regular basis. Inflammatory parameters and BASDAI were recorded at two-year followup. 

### 2.3. Study Assessments

The efficacy of therapy was determined by evaluating changes in MRI of the sacroiliac joints and by judging alterations in clinical and functional assessments. The Spondyloarthritis Research Consortium of Canada (SPARCC) MRI Spinal Inflammation Index was calculated [17]. Peripheral involvement was assessed by counting the number of swollen/tender joints out of a total of 28 joints; disease activity score 28 (DAS 28) [18] and Health Assessment Questionnaire (HAQ) [19] were recorded. The markers of inflammation (C-reactive protein (CRP), erythrocyte sedimentation rate (ESR), and hemoglobin) were recorded. 

The initial study endpoints were (1) to determine the proportion of responders at week 16 defined as ≥50% and/or 2 cm improvement from baseline of Bath AS disease activity score (BASDAI) [20] and (2) to determine the improvement at weeks 16 and 56 from baseline in the following parameters: BASDAI [21], spinal movement (Bath AS Metrology Index; BASMI) [22], spinal function (Bath AS Functional Index; BASFI) [23], patient's global assessments (BASG) [24], inflammation in the sacroiliac joints as measured by MRI- and health-related quality of life (HRQoL) assessed using the Short Form- (SF-) 36 questionnaire [25, 26]. The second endpoint of the study was to evaluate the treatment effect maintenance following infliximab dose reduction during the second year by assessing the laboratory inflammatory parameters and AS disease activity (BASDAI). 

### 2.4. Health Related Quality of Life

HRQoL was assessed with the Swedish version of the Medical Outcomes Study (MOS) 36-Item Short Form Survey (SF-36) at baseline and at weeks 16 and 56. The questionnaire consists of 36 questions on different dimensions of quality of life and global health, including physical function (PF), role-functioning (RP), bodily pain (BP), general health (GH), vitality (VT), social functioning (SF), role-emotional (RE), and mental health (MH). The first four variables are summarized into a physical component score (PCS) and the last four into a mental component score (MCS). The scores of the different dimensions range between 0 and 100, with 100 corresponding to the best possible health and zero to the worst conceivable self-perceived health. The PCS and the MCS are standardized to a mean (SD) value of 50 [25–27]. A randomly chosen sex and age-matched reference group of healthy controls (*n* = 528) from the Swedish SF-36 national normative database (*n* = 8930) was used for comparison [28].

### 2.5. Statistical Analysis

Non-parametric statistical methods were employed due to small sample size. The data is presented as medians (25th–75th percentiles). The Mann-Whitney *U* test was used to calculate changes between patient variables and controls in the SF-36 test. Fisher's exact test was employed to calculate changes at different time points with respect to percentage. Comparisons between measures at different time points were calculated with Friedman test and thereafter the two-tailed Wilcoxon signed rank test for paired samples was employed to calculate changes within the treatment groups. Associations between variables were assessed by Spearman's rank correlation test and coefficient expressed as Spearman's rho. Patients who dropped out were included in calculations until withdrawal. For the analysis of the primary and secondary outcome parameters, data from the 19 and 18 patients who had completed the study at 16 and 56 weeks, respectively, were used. For the third endpoint, data was available for 15 patients. Analyses were performed using Stat View version 5.0.1 for Microsoft Windows. A *P* value < 0.05 was considered statistically significant.

## 3. Results 

### 3.1. Patient Demographics and Baseline Characteristics

**Figure 1:**
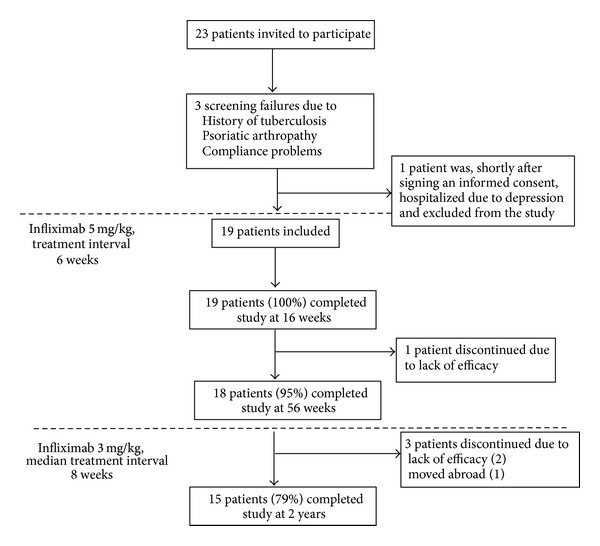
Flow chart of patient inclusion and dropouts.

Out of 23 patients invited to participate, 3 were screening failures (Figure 1). One patient dropped out shortly after inclusion due to depression demanding hospitalization and was therefore excluded from the study and analysis. Nineteen patients were included in the study and analysis. Fifteen (79%) completed the study after 2 years (Figure 1). 

All patients were HLA-B27 positive and fulfilled the modified New York criteria [16] for AS with radiological confirmation. Seven patients (37%) had a history of uveitis and eight patients (42%) had peripheral joint involvement. The patients' characteristics are shown in Table 1. 

At inclusion, 18 patients received medication with NSAIDs and one patient was treated with 5 mg prednisolone that was gradually tapered out within the first weeks. At the time of infliximab treatment initiation, all patients were also receiving a low dose methotrexate (median dose 7.5 mg/week). The purpose of adding MTX to the treatment regimen was mainly to improve the treatment effect trying to prevent the antidrug antibody formation against infliximab. 

### 3.2. Clinical Treatment Response during the First Year

The proportion of responders in the study defined as ≥50% or 2 cm improvement at week 16 from baseline of BASDAI was 16 (84%) patients. Significant reduction in disease activity as assessed by BASDAI index (*P* = 0.0002) was observed both at weeks 16 and 56 in comparison with baseline (Table 1, Figure 3), as well as significant increase in spinal functional activity as assessed by BASFI score (*P* < 0.0004) along with significant decrease in spinal metrological measures (BASMI, *P* < 0.007). CRP was elevated in 74% of patients at study start and decreased significantly along with ESR, whereas increase in hemoglobin levels was observed (Table 1). At 16-week followup, 10 patients (53%) required regular concomitant use of NSAIDs as compared to 18 (95%) at baseline (*P* = 0.0078). DAS28 decreased significantly already 16 weeks after treatment initiation (*P* = 0.0037) and none of patients displayed peripheral arthritis at week 56. 

### 3.3. The Effect of Infliximab Dose Reduction on Disease Activity

During the second treatment year the dosage of infliximab was reduced to 3 mg/kg and the infusion interval extended to every 8 weeks. Laboratory inflammatory parameters and BASDAI were recorded. After a significant reduction between baseline and weeks 16 and 56, the clinical signs and symptoms did not significantly change during the second year of treatment. No significant increase in BASDAI (median 2.1 (IQR 0.6–3.6) versus 3.2 (0.4–4.2), ns) was observed. The increased ESR and CRP at baseline normalized following treatment with infliximab after 16 weeks and were sustained throughout the study period of 56 weeks and 2 years. Importantly, patients did not have any greater need for NSAIDs or analgesics after the infliximab dose reduction (Table 1). 

### 3.4. Radiologic Findings

**Figure 2:**
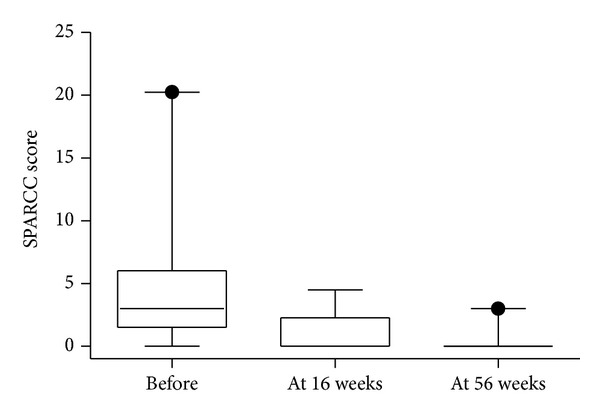
Change in SPARCC scores over time. Box plots show the 25th and 75th percentiles. Horizontal solid lines within boxes indicate medians and vertical bars indicate the 5th and 95th percentiles.

**Figure 3:**
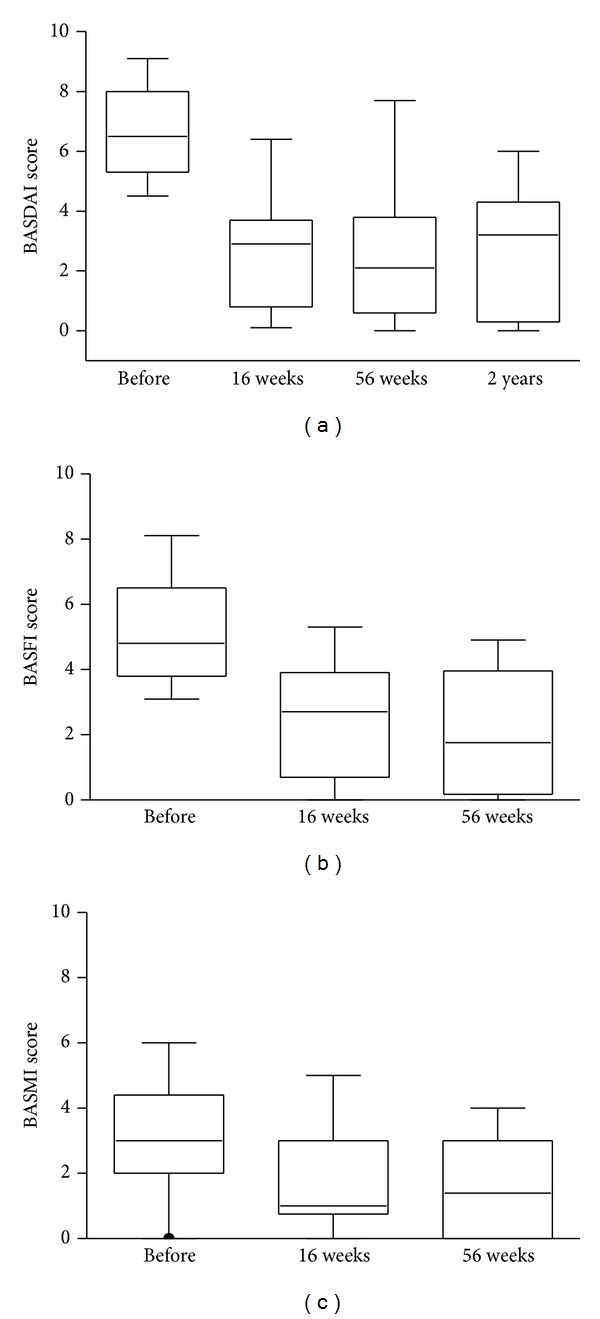
Change in BASDAI, BASFI, and BASMI scores over time. Box plots show the 25th and 75th percentiles. Horizontal solid lines within boxes indicate medians and vertical bars indicate the 5th and 95th percentiles.

**Figure 4:**
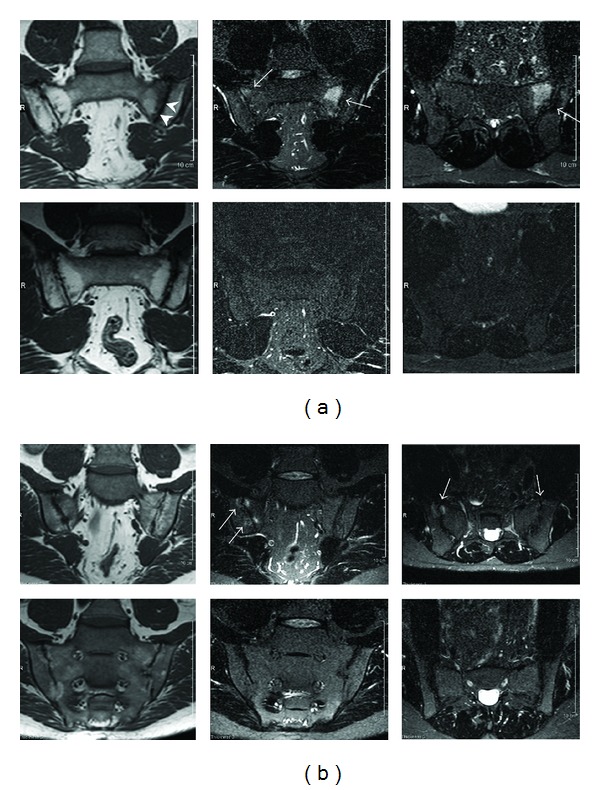
(a) A representative MRI study at inclusion (top row) with bilateral inflammatory changes (arrows). There is a 2 × 3 cm high signal intensity lesion on the sacral side of the left sacroiliac joint on the STIR images (coronal and axial) with corresponding low signal intensity on the coronal T1-weighted image. At the right sacroiliac joint, there are smaller and more subtle lesions on both sides of the joint. There are sclerotic changes along the left joint (arrowheads). At 16 weeks (bottom row), no oedematous lesions can be seen. (b) An MRI study showing bilateral sacroiliitis in an AS patient at inclusion and at 16 weeks. At inclusion, there are bilateral inflammatory changes on the MRI images (top row, arrows). At the right sacroiliac joint, there is a 1 × 1 cm high signal intensity lesion in the sacrum on the STIR images (coronal and axial) with corresponding low signal intensity on the coronal T1-weighted image. On the left, there are smaller and more subtle lesions on both sides of the joint. There are sclerotic changes along the right sacroiliac joint. At 16 weeks (bottom row), no oedematous lesions can be seen.

All patients had NY criteria unilateral grade 3 sacroiliitis or worse on preinclusion radiological studies. Four had bilateral ankylosis and one had unilateral ankylosis with contralateral grade 3 sacroiliitis. Eleven patients had bilateral grade 3 sacroiliitis; three patients had grade 3 on one side and grade 2 sacroiliitis on the other. All four patients with radiological bilateral ankylosis had bilateral bone marrow oedema at inclusion. MRI of the sacroiliac joints showed signs of inflammation at baseline in 15 patients (75%), with median SPARCC score 3.75 (IQR 2.25–9). The MRI-defined inflammatory changes in the sacroiliac joints decreased significantly (*P* = 0.0012) after initiation of infliximab therapy in these 15 patients (median decrease in SPARCC score −2.25, IQR −1.5–6) and disappeared in 10 patients (67%) at 16 weeks (Figures 4(a) and 4(b)). At 56 weeks, two patients still had MRI-verified inflammatory changes. The change in SPARCC scores over time in the whole study group is shown in Figure 2.

### 3.5. Health Related Quality of Life

**Figure 5:**
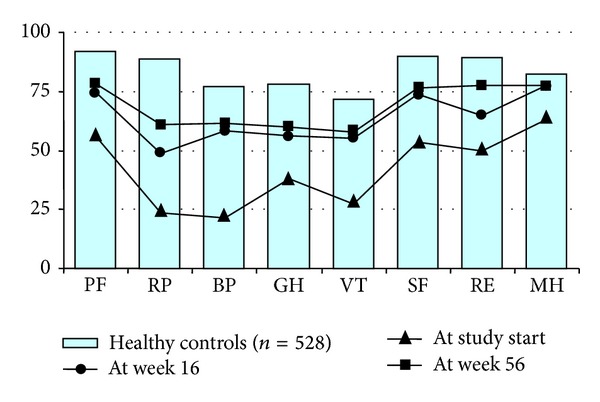
The patients' health related quality of life measured by SF-36. The graph shows the mean scores for the eight SF-36 domains for the normal population and the patients with AS at baseline, at 16 weeks, and at 56 weeks. A significant improvement in patients' health related quality of life was observed at weeks 16 and 56 as compared with baseline.

At baseline, the patients scored their quality of life significantly worse (*P* < 0.0001) than the age-matched reference population on all SF-36 subscales and their component summary scores were significantly lower (*P* < 0.0001) as compared to controls (Table 1 and Figure 5). A significant increase in patients' health parameters was observed at week 16 as compared with baseline values regarding physical function (PF; *P* = 0.01), bodily pain (BP; *P* = 0.001), general health (GH; *P* = 0.02), vitality (VT; *P* = 0.026), and social functioning (SF; *P* = 0.046). By week 56 after induction of infliximab treatment, the patients' quality of life had significantly improved also regarding their functioning role (RP; *P* = 0.005) and mental health (MH; *P* = 0.025). Importantly, at week 16 the AS patients had reached an MH status comparable with age-matched reference population and by week 56 no differences could be observed between the groups regarding their MH or emotional role (RE) (Figure 5). 

## 4. Discussion

In the present pilot study, we evaluated the clinical effect of infliximab treatment (5 mg/kg) on disease activity, patients' quality of life, and MRI-defined inflammatory changes in the sacroiliac joints in patients with HLA-B27 positive active AS. In addition, we explored whether the clinical treatment effect achieved with a standard dose of 5 mg/kg of infliximab every 6 weeks was maintained after a dose reduction to 3 mg/kg every 8 weeks. 

It is currently recommended that infliximab 5 mg/kg body weight should be administered every 6 weeks to treat AS and most randomized, placebo-controlled studies have evaluated this treatment regime [29]. Recently, a few reports have been published indicating that infliximab, when initiated in a lower dose as commonly used for treatment of rheumatoid arthritis (3 mg/kg at 8 weekly intervals), was also effective in decreasing inflammatory symptoms of active AS, although increased doses are needed in 15%–62% of patients according to results obtained from different studies [10–15]. To date, there is no published data regarding whether infliximab treatment effect is also maintained after dose decline and extension of infusion frequency. We observed in our pilot study that infliximab dose reduction from 5 mg/kg to 3 mg/kg along with longer infusion interval during the second year maintained the treatment effect and no significant increases in inflammatory parameters and/or BASDAI were seen. 

Although our study population consisted of HLA-B27 positive AS patients with long disease duration, the initial endpoint of the study at 16 weeks was fulfilled in 84% of patients, the treatment response being surprisingly high. After 12 weeks of treatment with infliximab, 53% of patients achieved >50% of reduction in BASDAI score as first reported by Braun et al. [30]. In the ASSERT trial, the respective improvement in BASDAI score from baseline to week 24 was achieved by 51% of patients [5]. It should be emphasised that all our patients concomitantly received methotrexate (median dose 7.5 mg/week) during the study period irrespective of the presence of axial or peripheral joint involvement. There are scarce data and conflicting evidence for the efficacy of DMARDs, including methotrexate, for the treatment of AS [31]. Pérez-Guijo et al. reported in their study with 19 participants a greater reduction of BASDAI score with combination of infliximab-methotrexate therapy as compared with infliximab alone [32]. However, these findings could not be confirmed in studies by other authors [33, 34]. Most studies indicate no evidence for the efficacy of methotrexate for the treatment of axial disease [4] and there is yet not enough support to suggest a beneficial effect of methotrexate in AS patients with mainly peripheral joint involvement [31, 35]. Since 42% of our study patients displayed signs of peripheral arthritis at study initiation, it is plausible that improvement of peripheral arthritis, supported by significant decrease in DAS28, could count for our high treatment response. Although patients were treated with high dose infliximab, the concomitant administration of methotrexate might possibly have some additive effect on the therapeutic response. However, it should be emphasised that the purpose of adding MTX to the treatment regimen was mainly to improve the treatment effect by preventing the antibody formation against infliximab, not the genuine anti-inflammatory effect. Infliximab is a monoclonal anti-TNF antibody of IgG subtype and has the potential to cause cell lysis while binding to the specific antigen on the cell surface [36]. Along with this antibody-dependent cell-mediated cytotoxicity reaction, infliximab has been suggested to cause alterations in apoptosis and thereby reveal nuclear antigens to the immune system [36]. Infliximab is, due to its chimeric nature, immunogenic, and concomitant treatment with methotrexate might therefore decrease the formation of antidrug antibodies. We observed an appearance of anti-infliximab antibodies at the study end in 4 out of 15 (27%) patients. Concomitant administration of methotrexate might therefore possibly enhance the efficacy of infliximab in AS, by preventing anti-infliximab antibody formation. Of note, since the patients were tested for antibodies at the study end, the data regarding anti-infliximab antibodies in drop-out patients is unavailable. Infliximab treatment decreases disease activity and inflammatory parameters from the very beginning of treatment and our results are consistent with previously published studies [37–39]. In accordance with the clinical results in the current study, treatment with infliximab resulted in a significant reduction of inflammatory changes of the sacroiliac joints as depicted by MRI using the SPARCC scoring system [17, 40]. We also observed that the efficacy of infliximab for improving the clinical signs and symptoms of AS along with reduced sacroiliac joint inflammation is associated with significant improvement in patients' health related quality of life. As shown by SF-36, substantial and sustained improvements of several physical health domains were seen already after 16 weeks of infliximab treatment. Interestingly, in the current trial mental domains improved as well and no significant differences were observed regarding mental health as compared to healthy control population at week 16 or regarding mental health and role emotional at 56 weeks. However, in ASSERT trial and in subsequent 2-year followup of ASSERT cohort, the results regarding SF-36 mental health component in infliximab treated AS patients did not show significant improvement as compared to patients treated with placebo [5, 39].

There are limitations of this study that should be taken into consideration. The main shortcoming of this study is the relatively small number of included patients and that it is not a blinded randomised controlled study. However, to our knowledge this is the first pilot study describing maintained effect of infliximab dose reduction in treating patient with established active HLA-B27 positive AS with long disease duration in the clinical praxis. These observations are of clinical importance and open the opportunity to reduce the drug costs and improve safety profile since infliximab is expensive and carries higher risk for side effects. However, there is a need for a prospective randomised controlled trial with larger sample size to confirm our results.

In conclusion, the current study has shown that treatment with infliximab along with methotrexate in patients with active HLAB27 positive AS with long disease duration resulted in significant and rapid improvement, which was sustained during the course of the study despite reduced dose and extended infusion intervals. 

## Figures and Tables

**Table 1:** Clinical and laboratory characteristics of patients at baseline and following treatment with infliximab 5 mg/kg every 6 weeks (time points at 16 weeks and 56 weeks) and after continuing infliximab treatment 3 mg/kg every 8 weeks (time point at 2 years).

Characteristics	Baseline	16 weeks	56 weeks	2 years
Number of men/women	14/5	14/5	14/4	13/2
Age (mean years ± SD)	39 ± 9			
AS symptom duration (mean years ± SD)	13.1 ± 8.9			
AS diagnosis duration (mean years ± SD)	6.9 ± 7.0			
Peripheral arthritis, number (%) of patients	8 (42%)	2 (10%)	0 (0%)	0 (0%)
Uveitis, present or in history, number (%) of patients	7 (37%)	0 (0%)	0 (0%)	0 (0%)
MTX dose mg/week; median (IQR)	7.5 (7.5–9.4)	7.5 (7.5–7.5)	7.5 (7.5–7.5)	7.5 (7.5–7.5)
Concomitant NSAID use, number (%) of patients	18 (95%)	10 (53%)*	9 (50%)*	7 (47%)*
Concomitant regular use of analgesics, number (%) of patients	7 (37%)	7 (37%)	7 (38%)	3 (20%)
Bath AS scores; median (IQR)				
BASDAI	6.5 (5.4–8.0)	2.9 (1.0–3.7)***	2.1 (0.6–3.6)***	3.2 (0.4–4.1)**
BASFI	4.8 (3.8–6.4)	2.7 (0.7–3.9)***	1.8 (0.2–3.9)***	
BASMI	3.0 (2.0–4.3)	1.0 (1.0–3.0)*	1.4 (0–3.0)**	
BASG-1	7.5 (6.0–8.3)	3.3 (1.1–4.3)***	1.1 (0–4.2)***	
BASG-2	7.2 (6.3–8.8)	4.9 (2.5–6.5)**	1.6 (0.2–3.5)***	
ESR mm/h; median (IQR)	23 (14–37)	6 (2–10)**	5 (2–8)***	7 (4–12)**
CRP mg/L; median (IQR)^#^	27 (8–45)	8 (8–8)**	8 (8–8)**	8 (5–8)**
Hemoglobin g/L; median (IQR)	135 (132–139)	141 (136–156)*	143 (138–154)*	153 (142–157)**
Disease Activity Score—DAS28; median (IQR)	3.03 (2.73–3.76)	1.74 (0.84–2.50)**	1.9 (0.98–2.11)*	1.80 (1.44–1.87)*
MRI SPARCC score; median (IQR)	3 (1.5–6.0)	0 (0–2.25)**	0 (0)**	—
SF-36 health score; median (IQR)				
PCS— physical total score	29.2 (23.1–33.2)	38.9 (32.8–50.9)**	45.4 (33.5–54.9)**	—
MCS—mental total score	37.3 (26.7–48.6)	53.3 (32.6–58.9)^ns^	53.5 (46.1–56.1)^ns^	—
Anti-infliximab antibodies, number (%) of patients	0/19 (0%)	NA	NA	4/15 (27%)*

BASDAI: Bath AS Disease Activity Index; BASFI: Bath AS Function Index; BASMI: Bath AS Metrology Index; BASG1: Bath AS Patients Global score (last 2 weeks); BASG2: Bath AS Patients Global score (last 6 months); CRP: C-reactive protein; ESR: erythrocyte sedimentation rate; HAQ: Health Assessment Questionnaire Disability Index; DAS28: Disease Activity Score; NA: not analyzed.

^#^Lowest detection limit for CRP <8 mg/L.

Comparisons between continuous measures at different time points as compared to baseline values were calculated employing the Wilcoxon signed rank test for paired samples and comparisons between the groups regarding percentage were calculated using Fisher's exact test. The level of significance is expressed as follows: *P < 0.05, **P ≤ 0.005, ***P ≤ 0.0005, ns: not significant.
